# Microbial Flora and Food Borne Pathogens on Minced Meat and Their Susceptibility to Antimicrobial Agents

**DOI:** 10.4314/ejhs.v20i3.69442

**Published:** 2010-11

**Authors:** Haimanot Tassew, Alemseged Abdissa, Getenet Beyene, Solomon Gebre-Selassie

**Affiliations:** 1 Department of Laboratory Sciences and Pathology, College of Public Health and Medical Sciences, Jimma University; 2 Department of Microbiology, Immunology and Parasitological, Faculty of Medical Sciences, Addis Ababa University, Ethiopia

**Keywords:** Food pathogens, Staphylococcus aureus, Minced meat

## Abstract

**Background:**

Food-borne pathogens are the leading cause of illness and death in developing countries. Changes in eating habits, mass catering, unsafe food storage conditions and poor hygiene practices are major contributing factors to food associated illnesses. In Ethiopia, the widespread habit of raw beef consumption is potential cause for food borne illnesses. The present study aimed at investigating the microbial quality of meat available in common retail shops, restaurants and abattoir of Jimma City and determining susceptibility pattern of bacterial isolates.

**Method:**

A total of 165 samples from food establishments, butcher shops and a slaughter houses were processed and analyzed for the presence of indicator bacterial and potential food pathogens using standards methods. Antimicrobial susceptibility test was performed for Salmonella, Shigella and Staphylococcus aureus isolates using Kirby-Bauer disk diffusion method.

**Results:**

A total of 165 samples were collected from twenty four hotels and five butchers and an abattoir. Various food borne pathogens were isolated in 13 (43.3%) and indicator organisms in 29 (96.7%) out of the thirty food establishments (hotels, butchery and abattoir). Overall, ten different bacterial species were isolated which included, proteus spp 89 (53.9%), E. coli 44 (26.6%), Providencia spp 23 (13.9%) Citrobacter spp 15(9%), Pseudomonas spp 9 (5.5%), Klebsiella spp 2 (1.2%), Enterobacter spp 2 (1.2%), Salmonella spp 2(1.2%), and Shigella species 1 (0.6%). Out of the 44 E. coli isolates 37 (84%) were thermo tolerant E. coli and out of the gram positive organisms identified 20 (12.1%) were Staphylococcus aureus isolates. From the two Salmonella isolates one was susceptible against all 12 tested antimicrobials, while the other to all the 11 except cephalexin. Shigella dysentery was resistant only to co-trimoxazole and tetracycline. Out of the 20 S. aureus isolates, 90% showed resistance to oxacillin, 85% to ampicillin, 65% to erythromycin, 60% to amoxicillin, 35% to streptomycin, and 20% to vancomycin and all isolates were sensitive to co-trimoxazole (100%). In this study, 90% (18/20) of the S. aureus isolates were Methicillin Resistant Staphylococcus aureus.

**Conclusion:**

In this study high percentage of indicator organisms as well as food borne pathogens were identified, which shows unhygienic condition of handling and processing in the food establishments. Our data also confirmed the presence of resistant food pathogens; particularly Staphylococcus aureus isolates which are Methicillin Resistant Staphylococcus aureus and multidrug resistant that emphasizes close follow up in the utilization of antibiotics. Therefore, meat handlers and sellers should be educated on the adverse effect of lack of proper personal, environmental hygiene and sanitation. In addition, consumers should be made aware of the risk of consuming raw and inadequately cooked meat.

## Introduction

Food-borne pathogens are the leading causes of illness and death in developing countries resulting in the lose of labor force which could have contributed in the economic growth (1). Changes in eating habits, mass catering, unsafe food storage conditions and poor hygiene practices are major contributing factors to food associated illnesses (2). Contaminated raw meat is one of the main sources of food-borne illnesses (3, 4). The risk of the transmission of zoonotic infections is also associated with contaminated meat. International food management agencies, especially the World Health Organization (WHO), the Food and Agriculture Organization (FAO) and the International Hazard Analysis Critical Control Point Alliance (HACCP) have already provided guidelines to member countries about safe handling procedures such as HACCP and Good Manufacturing Practices (GMPs).

In Ethiopia, the widespread habit of raw beef consumption is a potential cause for food borne illnesses besides, the common factors such as overcrowding, poverty, inadequate sanitary conditions, and poor general hygiene (5). Raw meat is available in open-air local retail shops without appropriate temperature control and this is purchased by households and also minced meat (Kitfo) is served at restaurants as raw, slightly-cooked or well- cooked.

Meat processing at retail level is likely to contribute for the higher levels of contamination in minced beef as compared to carcasses (6). The presence of even small numbers of pathogens in carcass meat and edible offal may lead to heavy contamination of minced meat when it is cut into pieces; as more microorganisms are added to the surfaces of exposed tissue (7).

Previous studies conducted in Addis Ababa indicated the occurrence of pathogens including Salmonella in different food animals, meat and meat products. In addition, outbreaks of infections somehow related with poor hygiene and consumption of contaminated food were reported in Ethiopia (8) and some were caused by Salmonella and Shigella (6, 9). Moreover, antibiotic resistance levels are also elevated among food-borne pathogens such as Salmonella and Shigella (8, 10). Although, it is difficult to prove a direct role of drug resistance in bacteria contaminating food items with increased clinical cases of resistant infections, the presence of such bacteria in food items could play a role in the spread of antimicrobial resistance amongst food-borne pathogens (11).

Thus, adequate information should be gathered to develop an effective strategy to reduce the outbreak of food born illnesses and resistance burden in the community. This study therefore aimed at investigating the microbial quality of meat available in common retail shops, restaurants and abattoir of Jimma Town and to determine susceptibility pattern of bacterial isolates.

## Materials and Methods

This study involved randomly selected 24 hotels and 5 butcher shops and an abattoir in Jimma Town from April to August 2009. Approximately six samples were collected from each outlet. Accordingly, a total of 120 minced meat (‘kitfo’) samples from 24 food establishments were collected. Twelve environmental samples from the slaughter house were collected comprising surface swabs taken from 30 cm2 of the surface of meat-cutting equipment such as knives, wooden boards and weighing scales. In addition, 25 and 8 carcasses swab samples comprising an area of 100 cm2 lean meat were collected from the butcher shops and slaughter house, respectively. The samples were collected in a sterile container and immediately transported using ice box to the laboratory for bacteriological analysis. The 24 food establishments were categorized in to three (A, B, C) groups according to the standards set by the town municipality. A hotel in standard “A” fulfils: septic tank, waste pit, standard toilet (separate for male and female), standard kitchen, water tanker, medical checkup for workers every three months, clean kitchen utensils, standard bed room, and fire extinguisher. A hotel in standard “B” has all in “A” but lacks water tanker, medical checkup for workers every six months and no fire extinguisher; whereas, a standard “C” hotel has all defects seen in “B” and lacks septic tank, waste pit and has only one room toilet.

A 25gm sample of the minced meat was homogenized in 225 ml of buffered peptone water (BPW) using homogenizer. The final homogenate gave a 1:10 dilution and was incubated for 24hrs at 350C. Out of this a serial dilution of 1:106, 1:107 and 1:108 was prepared and used for further analysis. All media and antibiotic discs were purchased from Oxoid, UK.

For isolation and identification of S. aureus a loop full of sample from the homogenate were inoculated on Manitol salt agar (MSA) and golden yellow colonies on MSA which were catalase positive and coagulase positive isolates were identified as S. aureus. Next, the enriched sample was inoculated on MacConkey and xylose lysine deocycholate agar (XLD) and incubated at 37oC for 24 hours. For isolation and identification of Salmonella the following steps were followed. The sample was enriched on tetrathionate (TT) and Rappaport Vassiliadis (RV) broth selective media. Then the gram-negative organisms including Salmonella and Shigella were identified using a battery of biochemical tests like, reactions on Kligler's iron agar (KIA), lysine iron agar (LIA), urea, motility test, simmon's citrate, oxidase, indole and mannitol fermentation tests. To confirm thermo tolerant E. coli; suspected isolates were incubated at 440C water bath for 48 hours in nutrient broth and they were tested for growth at 440C and checked for indole production. Isolates which demonstrated growth at 440C with indole production were confirmed as thermotolerant E. coli. Furthermore, Salmonella and Shigella strains were sero-grouped by slide agglutination tests using poly O and single O-groups antisera (Remel, Europe Ltd, UK).

Antimicrobial susceptibility testing for Salmonella, Shigella and S. aureus was performed using the disk diffusion method and results were interpreted using the criteria of the National Committee for Clinical Laboratory Standards (NCCLS, 2000). The antibiotics used were Ampicillin (A-10µg), Amoxicillin/clavulanic acid (AM-10µg), Chloramphenicol (C-30µg), Streptomycin (S-10µg), Trimethophrim (Tr-5µg), Tetracycline (T-30µg), Ciprofloxacin (Cf-5µg), Nalidixic acid (Na-30µg), Gentamicin (G-10µg), Kanamycin (K-30µg), Co-trimoxazole (SXT-25µg) and Cephalexin (Cp-30µg). The criteria used to select the antimicrobial agents tested were based on the availability and frequency of prescription for the management of bacterial infections in Ethiopia. Antibiotic susceptibility testing for S. aureus was determined for Ampicillin (A-10 µg), Amoxicillin/clavulanic acid (Am-10 µg), Erythromycin (E-15 µg), Oxacillin (Ox -1 µg), Streptomycin(S-10 µg), Co-trimoxazole (SXT-25 µg) and Vancomycin (Va-30 µg).

A standard reference strain of E. coli (ATCC 25922), sensitive to all antimicrobial drugs was used as a quality control for disk diffusion.

Data obtained from this study was entered to computer, analyzed using SPSS for windows version 16.0 and interpreted. Ethical approval for the study was obtained from the Institutional Review Board (IRB) of Faculty of Medicine, Addis Ababa University and from Jimma University Ethical Review Board.

**‘Indicator organisms’** are defined as large group of bacteria including certain pathogenic bacteria, which are relatively easy to measure as a group and the presence of this in food, is likely to indicate the presence of pathogenic bacteria.

**‘Pathogenic bacteria’**- bacteria that are known to cause infectious diseases in human.

## Results

A total of 165 samples from 29 food establishments and a slaughter house were analyzed. Of the 30 establishments (24 hotels, 5 butcheries and an abattoir), various food borne pathogens in 13 (43.3%) and indicator organisms in 29 (96.7%) of them were isolated. Indicator organisms were isolated from all the 5 butcheries and food pathogens from 3 (60%) of them (Table 1).

**Table 1:** Number of pathogenic and indicator organisms isolated from different food establishments, butcher shops, and abattoir in Jimma City, Southwest Ethiopia, 2009.

Sample source	Food establishments involved in the survey	Food establishments positive for pathogenic organisms	Food establishments positivefor indicator organisms
Hotels			
A	6	2 (33%)	6 (100%)
B	13	5 (39%)	12 (92%)
C	5	2 (40%)	5 (100%)
Butcher shop	5	3 (60%)	5 (100%)
Abattoir	1	1 (100%)	1 (100%)
Total	30	13 (43.3%)	29 (96.7%)

Key: A, B, C are food establishments categorized according to their standards set by the Jimma town municipality (see the result section).

The only abattoir in Jimma Town was included in the survey and 20 samples were collected from various locations and meat processing instruments. Out of these 20 samples, 4(20%) were found to have pathogenic organisms while 16 (80%) carried indicator organisms (Table 2).

**Table 2:** Number of pathogenic and indicator organisms isolated from abattoir, Jimma Town, southwest Ethiopia, 2009.

Sample source	Samples collected	Samples positive for pathogenic organisms	Samples positive for indicator organisms
Carcasses	8	1 (12.5%)	7 (87.5%)
Knives swab	8	3 (38.0%)	6 (75.0%)
Floor swab	4	0(0%)	3 (75.0%)
Total	20	4 (20.0%)	16 (80.0%)

Out of the 165 samples collected, ten different bacteria were isolated. Most of the isolates were members of the Entrobacteraeceae family including proteus spp 89 (53.9%), E. coli 44 (26.6%), Providencia spp 23 (13.9%), Citrobacter spp 15(9%), Pseudomonas spp 9 (5.5%), Klebsiella spp 2 (1.2%), Enterobacter spp 2 (1.2%), Salmonella spp 2(1.2%), and Shigella group-A 1 (0.6%).

**Figure 1:**
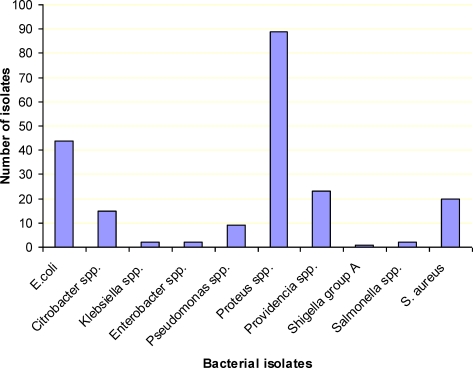
Frequency distribution of bacterial isolates from samples (minced meat, unprocessed meat and from abattoir), Jimma town, southwest Ethiopia, April – August, 2009.

Out of the forty four E. coli isolates 37 (84%) were thermo-tolerant E. coli. Of the gram positive organisms, identified 20 (12.1%) were Staphylococcus aureus isolates (Figure 1). The two Salmonella isolates were in group A (isolated from minced meat) and group B (from the butcher's shop).

Of the two Salmonella isolates, group A was susceptible to all the 12 drugs whereas, group B was susceptible to the eleven tested drugs but resistant only to Cephalexin. The single Shigella isolate was susceptible for Ampicillin, Amoxicillin, Ciprofloxacin, Naldixic acid, Gentamicin, Kanamycin, Chloramphenicol and Cephalexin but resistant for Co-trimethoxazole, Tetracycline, Streptomycin and Trimethophrim.

A total of 20 S. aureus isolates were tested for seven antibacterial agents which showed 90% were resistant to Oxacillin, 85% to Ampicillin, 65% to Erythromycin, 60% to Amoxicillin, 35% to streptomycin, and 20% to Vancomycin but all (100%) of the isolates were sensitive to Co-trimoxazole. Eighteen of the 20 (90%) S. aureus isolates were Methicillin Resistant Staphylococcus aureus (MRSA). However, 2(10%) of the 20 S. aureus isolates were sensitive to all antibiotics tested (Table 3).

**Table 3:** Resistance pattern of S. aureus, Jimma town, southwest Ethiopia, April – August, 2009.

Antimicrobials	Resistant isolates Number (%)
Ampicillin	17(85)
Streptomycin	7 (35)
Amoxacillin	12(60)
Erythromycin	13(65)
Oxacillin	18(90)
Vancomycin	2(10)
Co-trimoxazole	0(0)

The resistance pattern varied among five drugs and in general S. aureus showed high level of resistance to Ampicillin, Amoxicillin, Oxacillin, Erythromycin and Streptomycin. From the 18 organisms tested 15 exhibited resistance to three or more antibiotics (Table 4).

**Table 4:** Antibiogram of Staphylococcus aureus isolates from different food establishments, Jimma town, south west Ethiopia, 2009.

Antibiotics	No and % resistant	S. aureus
	no	%
a	1	5
A, Ox	1	5
AM, E	1	5
A, E, Ox	1	5
A, Am, Ox	1	5
A, S, Ox	1	5
AM, Van, Ox	2	10
A, AM, E, Ox	5	25
A, E, S, Ox	4	20
A, AM, E, S, Ox	1	5

Total	18	90%

A = Ampicillin, E = Erythromycin, AM = Amoxicillin, S = Streptomycin, Ox = Oxacillin, Va = Vancomycin

## Discussion

Food borne illnesses caused by non- typhoid Salmonella, S. aureus and E. coli represents a major public health problem worldwide. These pathogens are transmitted mainly through consumption of contaminated food and the presence of these organisms in meat animals and in raw meat products has relevant public health implications (12). In this study, it was found out that almost all of the food establishments had pathogenic and indicator bacteria. Similarly, pathogenic and indicator bacteria were isolated from one-fifth and four-fifth of the samples collected from the abattoir, respectively. This finding shows how high the magnitude of contamination at food establishments and at slaughter houses which may contribute to the incidence of food associated illnesses.

In this study, E. coli was isolated in the majority of food establishments, which indicates the presence of unhygienic food processing. Moreover, the identification of thermo-tolerant E. coli showed the presence of recent fecal contamination (13). This is in agreement with the study done in Trinidad meat processing plant where the isolation rate was 90% of E. coli from raw meat and 34.4% of E. coli from unprocessed ready-to-eat product (14). Similarly, a study in Tehran Restaurants 27(12.5%) E. coli were isolated from grilled ground meat (15). Naturally, high frequency of E. coli in minced meat shows high rate of contaminations during processing. Thus, E. coli's presence in minced meat might have originated from animal tissues or contaminated tools used in slaughtering and related treatment or cutting process. Although, serotyping of Escherichia coli isolates was not done in this study, isolation of these bacteria should be taken as a considerable threat (16).

As studies conducted elsewhere showed the importance of Proteus as an indicator of unhygienic food processing practice (17), the high frequency of Proteus species isolation observed in this study should be considered seriously. Proteus was also isolated from food sample and in stools of patients with gastroenteritis (18). Recently there was an outbreak report from Beijing which was attributed to contaminated food and Proteus mirabilis was identified as causative agent (19). Thus, the role of Proteus as food pathogen should be further investigated.

The identification of Providencie spp, Cirobacter spp and Pseudomonas spp in food in this study appears not to be a threat to public health, however, they could be opportunistic pathogens to immuno-compromised and debilitated individuals (18).The identification of only two Salmonella isolates in this study is much lower than other reports where the isolation rate was 20% in Gaborone, Botswana 20% (20) and 9% in Awasa, Ethiopia (21). Probably, low Salmonella detection could be due to the fact that Salmonella usually contaminates chicken and water than beef. The other factor may be the low prevalence of Salmonellosis in the Jimma community as recently has been reported (22).

The existence of Salmonella group A in minced meat indicates that the contamination is of human origin and result of poor personal hygiene during handling and processing of food. The presence of Salmonella group B indicates the contamination is from both sources for this group comprises many strains of Salmonella species (23). Only one Shigella dysentery was isolated from the raw minced meat sample, similar to another report from Ethiopia which identified only 3(2%) Shigella spp. from 150 Macaroni samples studied (21).

The high prevalence rate (12%) of Staphylococcus in unprocessed slices of meat and abattoir indicates the presence of cross contamination, which is usually related to human skin and clothing. As the carrier rate varies with different populations ranging from 10 to 40% in adults outside the hospital environment, Carriage may be intermittent or continuous over a long period of time. Approximately, 92% of people demonstrated nasal carriage of S. aureus in Jimma town (24) and this level of food contamination by this pathogen may lead to food intoxications. This pathogen is a major target in the screening of slaughterhouse carcasses to monitor hygienic conditions (25). Therefore, this study shows the slaughter house is unhygienic and needs close follow up to improve the situation.

In this study, one of the two Salmonella strains isolated has been found to be sensitive to all twelve antibiotics while the other showed resistance to only Cephalexin. This observation is comparable to study conducted in Addis Ababa which showed eight of the ten Salmonella isolates were sensitive to all ten drugs tested and the remaining two were resistant to Sulphadiazine (26).

The MRSA and multi drug resistant S. aureus in this study is higher than the one reported in a previous study in Jimma where 52% MRSA and 72% of multi drug resistant (27). The presence of 90% MRSA strain is demonstrating the fast growing and alarming situation to the public health system and the community. Thus, it requires strong controlling system of the personal hygiene and educating food handlers about the basic ideas of processing and producing a safe food.

In conclusion, the presence of food pathogens and indicator organisms such as thermo-tolerant E. coli in most of the food establishments shows poor food handling and processing practices. In addition, high proportion of methicillin resistant S. aureus isolates indicate the importance of making periodic monitoring of food establishments. Therefore, meat handlers and sellers should be educated on the adverse effects of lack of proper personal, environmental hygiene and sanitation. In addition, consumers should avoid eating raw and inadequately cooked food.
